# Two Cases of Vesico-Vaginal Fistula Cured

**Published:** 1859-01

**Authors:** Warren Brickell

**Affiliations:** Professor of Obstetrics, New Orleans School of Medicine


*Two Cases of Vesico-Vaginal Fistula Cured.* By WARREN BRICKELL, M. D., Professor of Obstetrics, New Orleans School of Medicine.

About three years ago my surprise was excited by the observation of a Medical friend, to the effect, that not a case of Vesico-Vaginal Fistula had ever been cured in New Orleans—that not a few operations

* Galen means the true Celt, with "milk-white throat," "golden hair" (Virgil,)or "flaxen poll" (Claudian;) his "love of drinking and brawling, loud voice, fierce eyes, and haughty insolence; and his equally pugnacious blue-eyed wife, with her swollen neck and gnashing teeth, using her fists like catapults." (Ammianus Marcellinus.)

had been undertaken by different surgeons here, but no good results had ever ensued. Considering this rather a stigma on the profession of so large a city, where medicine, in all its branches, should be as well cultivated as in any other portion of the world and more especially since the operation of Sims had been known to the world, I determined to apply myself to the subject. The first thing was to get all the necessary instruments, and these I procured from Tiemann & Co., of New York. Then I must get a case on which to operate.

In a private Hospital in this city, I was allowed to operate on a young negro woman, from Red River, who was considered incurable. On examination, I found a very large fistula indeed—so large, that when the patient would lie on her back, the inverted bladder would protrude through the vulva. This was an ugly case for a beginner, but the subject was otherwise healthy, and I determined to try it. Suffice it to say, that at various times I operated, by the methods of both Sims and Bozeman; that is, I used the silver wire, (claimed as entirely original by Sims,) with and without the clamps, and I used the wire, together with Bozeman's "button." I failed signally—for causes that I will hereafter mention. I then tried the operation for closure of the vulva, and in this I failed too. The patient was now carried to Dr. Bozeman, of Montgomery, Alabama, and I see he has reported her *cured*. It gives me the greatest pleasure to say here, that for curing this case, Dr. Bozeman deserves the greatest credit, and no one could more willingly award him all praise than I now do. That it was a bad case, there can be no doubt, and when I abandoned her I thought her incurable; but with the experience I now have, I believe I could cure as bad a case—though I have since seen one which has proved too much for me, the whole vesico-vaginal septum being wanting.

My second case was an Irishwoman of this city, with a fistula fully an inch and a quarter long, (across the septum,) and when exposed to view nearly three quarters of an inch wide, and very high up. I operated on this woman three times, at intervals of about six weeks to three months, the sutures on the upper edge of the fistula passing through the posterior lip of the uterus, and at the end of six weeks after the third operation, the fistula was only the size of a crow quill. Of course, I expected to cure this case, and in order to insure this end, I determined to wait two or three months, and let the parts become firm and solid. At the end of four months, I called to operate, and, to my surprise, found that she was more than three months advanced in pregnancy. She was averse to having anything done now, as she was anxious to have a living child, (both she and her previous child having been butchered by a so-called medical man); but, as there was extensive cicatrization of the vagina, independent of the seat of operation, I advised premature delivery. All advice was useless, however, and she went to full term, and had a fine living child, but with the deplorable result of bursting the cicatrix in the seat of operation. A few weeks after delivery, I examined her, and found the fistula nearly as large as when I first undertook the case. She promised to submit to operation again as soon as her babe was old enough to spare her, and I still hoped to cure her. Of the three operations performed on her, one was with the wire and clamps of Sims, one with the wire alone, and one with the wire in conjunction with Bozeman's button. The clamp operation effected little, and the other two operations appeared to be followed by equally beneficial results. My object was to test all the best modes recommended.

*First Case Cured.*—On Christmas day, 1857, I received a very fine looking negro woman from Mr. Yeamans, of Vicksburg, Mississippi, said to be laboring under Vesico-Vaginal Fistula. If I am not mistaken, my friend, Dr. Harper, of Vicksburg, had seen her, and recommended Mr. Y. to send her to me.

I will not retain the reader with a minutiæ of her previous history. Suffice it to say, that she was a stout, athletic woman, in good general health, the mother of nine or ten children, and about thirty-five years of age. In her last confinement she had been attended only by an ignorant negro midwife, (like the midwives in general, the curses of the communities in which they are found,) and, from long detention of the head of the child in the pelvic cavity, sloughing of the vesico-vaginal septum had taken place, leaving a permanent fistula, now about three quarters of an inch long, and seated transversely across the septum, about midway between the neck of the bladder and the uterus.

On the 30th December, 1857, I operated with the wire suture alone, (I mean always, tying the wire with the shot of Sims); no disagreeable symptoms followed; urine flowed through the catheter only; and on the ninth day, I removed the sutures, finding an apparently perfect cicatrix in place of the fistula. Of course, I used the catheter for several days, to draw off the water, not wishing to allow either distension of the bladder, or to tax it with expelling its contents. Notwithstanding this, however, the very first time an accumulation of water took place, and she endeavored to expel it herself, rupture of the cicatrix was the unfortunate result. Reflection on the subject, and subsequent experience, however, have satisfied me that I did not denude the edges of the fistula sufficiently, and that my cicatrix was too frail.

As soon as the parts were all firmly healed and in good condition, I operated again, this time using Bozeman's button, with the wire and shot. On the ninth day, I removed the sutures, and perfect union had taken place. This time I had denuded the parts extensively. The woman is still perfectly well.

*Case Second Cured.*—Early in January, 1858, Mrs. Mary M., native of Ireland, came to me from St. Louis. I found her in my ward in the Charity Hospital one morning. She said that about a year before she had been delivered of twins, the labor resulting in the establishment of vesico-vaginal fistula. Early in November, 1857, she was carried by her husband to the Hospital in St. Louis, and there she remained during the month of November and December. Previous to entering the Hospital, one of the most prominent surgeons of St. Louis had operated on her once, and after entering the Hospital the same surgeon had operated three times, but with no beneficial result; on the contrary, she thought the fistula "much larger than ever." While in the Hospital, she encountered a woman who had been an inmate of the Charity Hospital, New Orleans, and who told her that I could cure her. On this vague information she sold her feather bed (for her husband had abandoned her and left her penniless) for nine dollars and took a steamboat for this city.

When I encountered her in my ward, she was one of the most miserable looking objects immaginable. She was not exceedingly emaciated, but she was pale and dejected, without hope and without courage; the urine all the time pouring down her limbs, and the parts excoriated. I examined her, and found a fistula one inch and a quarter, by measurement, across the vesico vaginal septum, and, as the patient assumed the position on the hands and knees, three quarters of an inch—a huge hole into the bladder, and through which the inverted bladder rolled through the vulva, so as at all times to be seen by merely separating the limbs a little. Indeed, it was only by passing a sound through the urethra, and with it pushing the bladder upwards, (thus preventing inversion,) that the fistula could be properly defined.

As unpromising as things appeared, I cheered her with assurances that every effort would be made to cure her—that, if she would be patient, I would never give up as incurable, inasmuch as worse cases were reported as having been cured. She was put on good diet, fur nished a good bed, etc., etc., and on the 17th of the same month was ready for operation.

I denuded the edges of the fistula extensively, (the consequence of which was very free Hemorrhage,) and then passed firm sutures through, freely embracing the posterior lip of the uterus, (the fistula being pretty high up.) Bozeman's button was then fitted on, and fastened down with the leaden shot, as usual. She bled but little after the operation was complete, the water ran by the catheter, and when, on the tenth day, the sutures were removed, perfect union had taken place, though at one end of the seat of operation the cicatrix appeared rather frail. It was a very small spot, however, and I hoped that it would grow stronger as contraction took place. For several days the catheter was used, and it was not until the fifteenth day that she was allowed to get out of bed, or to pass water naturally. She improved daily, but one morning when I visited my ward, I found her dejected again, and she told me that she was again "leaking a liitle." On inquiry I found that a little dispute had arisen between her and the nurse, a scuffle ensued, and the nurse pushed her down violently, the consequence of which was immediate leakage of urine from the vagina. On examination, I discovered a small hole about an eighth of an inch in diameter, and on the left extremity of the cicatrix. I put her to bed at once, placing her on her right side, and introducing a permanent catheter in the bladder—still hoping that the parts would heal firmly. In this, however, I was disappointed. The result was the establishment of a small fistula, from which there was constant slight leakage, though it was too small to allow the urine to flow from the bladder as fast as secreted, and the consequence was that she would pass considerable quantities of urine *per vias naturales,* and ten or twelve times during the twenty-four hours—the bladder being, of course, still quite irritated.

About this time I found myself encountering another obstacle. Mine was a *medical* ward, this was a *surgical* case. Under the technicalities of the Hospital, I could not operate again. But my patient had come all the way from St. Louis to be operated on by me especially; no other medical man in the city was making any effort to cure such cases; all efforts previously made here had failed; she declared no one else should touch her; and I determined not to abandon her on any terms. She was too poor to hire either apartments or a nurse, so I took her to my residence and placed her in comfortable quarters. On the 18th of March I operated on her again. I denuded the edges of the little fistula freely, thereby making the opening considerably larger, and passed through two silver wire sutures, fastening them with the perforated shot. On the ninth day I removed these sutures, and union was complete. I now write on the 22d of September. Day before yesterday my patient was in my office to see me.—She is as well as she ever was in her life—she is well in spite of every obstacle, real and factitious, that has been cast in her way. She is not only well. but she is making her own living, and may some day assist in erecting a Hospital which shall know no limits to the encouragement it gives to every medical man who aspires to the exaltation of his profession above the standard of a mere mode of gaining a living.

*Observations.*—In the treatment of case No. 1, I labored under great disadvantages. First, I had no experience in the matter of operating, as well as in the management of such cases after operation—which I find to be quite as important as the proper performance of the operation itself. The patient, herself, was anything but obedient, (on several occasions, I found her out of bed within three days after operations,) and she was so far removed from me, that I could not see her oftener than twice in twenty-four hours. To Dr. Bickham, Resident Surgeon of the Hospital, I am deeply indebted for his ready assistance, both during and after operations, but, like myself, he was inexperienced in the management of such cases. After my operation, my great trouble was to keep the catheter clear, and in every instance, I now believe, that my operation failed because the urine accumulated in the bladder—the catheter having been plugged up by the phosphatic deposit which was abundantly poured out of the bladder. Had I just such a case in my own yard now, I believe I could cure it, but I would not undertake it under anything like the disadvantageous circumstances as I have related. He who undertakes to cure a case of vesico vaginal fistula, must not deceive himself with the idea that the operation is the great point. To be sure this must be properly performed, but I care not how well it is done, it will fail if the subsequent treatment is not properly conducted. And herein consists the actual labor in these cases—labor, too, of the most disagreeable kind imaginable.

I confidently anticipate curing the second case mentioned, if she returns to me. She is a fine, healthy woman, and, with my present experience, I believe I can relieve her with half the labor I have already expended on her. I was bitterly disappointed in the ultimate result in her case. I could scarcely have imagined that one in her situation would have subjected herself to the chance of becoming pregnant. But there is no accounting for tastes.

Case third, the first cured, was certainly the most obedient patient I ever met. She never for a moment deviated from the rules laid down and I am satisfied that the cure was much facilitated on this account.

Case four was, also, very patient, but never exhibited the same utter submission to rules that case three did; consequently, I had a great deal more trouble with her. I mention these little matters for the benefit of those who may undertake the operation. It is certainly a most important point to instil the mind of the patient with the importance of utter submission to directions. I have never experienced any professional labor so disagreeable and unsatisfactory as that bestowed on an obstreperous patient of this kind.

*Mode of Operation.*—Just at this time, it would seem almost like walking over new incrustations consequent on volcanic eruption, to institute a comparison between the more popular operations of the day; the surface is still pretty hot, and wo be unto him who happens to break through! However, I have nothing original to claim, and may, therefore, be pardoned for expressing my honest convictions in relation to the proposed plans of others. I think I have tried the "clamp" of Sims and the "button" of Bozeman sufficiently often, and have watched their operation sufficiently closely, to be able to judge of their value. The clamp, I do not think I shall ever try again, because I agree with its author, that it is not essential to the cure, and unless a great deal of skill is exercised in its application, it is dangerous. In a few words, I believe the "clamp" will soon be obsolete. The "button" I have applied with a great deal of care, and I have tried to convince myself that its *modus operandi* is what its author claims for it, but I am obliged to say that I cannot concede it anything like the influence claimed. For one simple reason I shall always use it or something like it, viz: the plate interposed between the tissues and the shot used for tying the sutures, prevents the shot from becoming imbedded, and sometimes even encysted, in the tissues, which I have found rather a troublesome complication. That the concave plate or button exercises any influence in keeping the lips of the wound in apposition, I confess I am at a loss to conceive; my conception of the physical laws here involved does not lead me to award such an influence. The wires alone exert the power of adapting the denuded surfaces, the shot tie the wires most efficiently and simply, and the "button" keeps the shot from being imbedded in the tissues. As to the salutary influence claimed for the button, in keeping the secretions of the vagina from coming in contact with the wound, I cannot attach any importance whatever to that; the only substance that need give the operator any anxiety in this respect, is the urine, which is in direct contact with the inner edges of the wound. If the lips of the wound are well adapted, infiltration of urine is prevented; then what cause is there for anxiety about the passage of the vaginal secretion opposite? More than this: the button itself covers a certain portion of the vaginal surface, which portion is exhaling the same matter, and this matter is absolutely held by the button in contact with the wound. In a few words, I am satisfied that to the silver wire used, we chiefly owe our success in these operations, though I am not prepared to say that *silver* wire alone will answer the purpose. On the contrary, the success of Mettauer, of Virginia, proves conclusively that the leaden suture is quite reliable. With my present experience I would not like to exchange a good silver wire for a leaden one; I would prefer the perforated shot to simply twisting the suture, and I would use the silver plate or "button" to prevent the shot from becoming imbedded in the tissues. I would thus combine the operations of Sims and Bozeman, regarding the latter as a very useful modification of the former.

*Position.*—Position, both during and after the operation, is a matter of great importance. I prefer the position on the hands and knees during the operation, because I have found that a more complete exposure of the seat of operation can be obtained in this way than in any other. I do not believe, however, in the idea of Dr. Sims, that the vagina is opened by *atmospheric pressure.* There are many cases in which no view can be had of the os uteri in this position, without great assistance from the speculum, and I have seen no case wherein the operation could be performed without the speculum.

After the operation, I have found the position on the side the best that can be assumed by the patient, and, with efficient assistance, the patient can change from one side to the other without endangering the success of the operation. As the patient lies on one side, the attendant can more readily attend to cleansing and replacing the catheter, etc., etc.

Although I have been obliged to contrive things to meet the exigencies of my cases, I claim no originality. I have a bed with a hole in the mattress, under which is placed a chamber vessel. I use an ordinary female catheter, with a piece of india-rubber tubing, about a foot long, attached to it with adhesive strips; the urine flows through the catheter into the tube, and thence into the vessel under the bed—thus effectually protecting the person of the patient from contact with the urine, and keeping the bedding perfectly clean. I have faithfully tried Sims' catheter, but cannot get it to answer half so well as the ordinary female catheter, with a few extra holes drilled into the sides. This latter has a twine passed through its eyes, and this is made fast to another string passed around the hips of the patient. With this arrangement, the *inner* end of the catheter can be elevated or depressed at will, and the catheter can be made to penetrate the bladder to any desired depth.

*Phosphatic Deposits.*—I have been greatly annoyed by the phosphatic deposits in all my cases, but latterly found great relief from the free use of Benzoic Acid. This I have given internally, and have injected it into the bladder, and with very beneficial results. Sometimes it has entirely corrected the tendency to deposition of the phosphates; at other times only partially.

*Denudation and Passage of the Sutures.*—I have found that any other than free denudation of the lips of the fistula, is unreliable, and I do not spare the tissues where there is plenty at disposal. As to the passage of the sutures, instead of taking care to avoid penetration of the mucous membrane of the bladder, as eanestly advised by some writers, I take espeoial pains to pass the needle clear through. I thus know that I have a firm hold, and have yet to see the slightest evil results. On one occasion, when the operation had failed to effect union of the edges of the fistula, I allowed several sutures to remain penetrating the entire walls of the bladder, and not the least irritation occurred. All observation has taught me that the silver wire is harmless.

I have thus given a cursory history of the first case of this disease ever cured in New Orleans. While I was operating on my second case, Dr. Schuppert, of this city, operated on and cured one, which was the second ever cured here, and my St. Louis case is the third. A few years ago, one of our most respectable surgeons reported a case in this Journal, but the fistula was re-established a short time afterwards. I am deeply indebted to many of my medical friends, both graduates and undergraduates, for their valuable assistance in my operations. They have worked hard with me, and often have I seen skepticism and disappointment on their faces when I would report failures. But we have succeeded at last, our path is now comparatively clear, and I hope to call on them again.—*N. O. Med. News & Hospital Gazette.*

